# Heterogeneity of Alkane Chain Length in Freshwater and Marine Cyanobacteria

**DOI:** 10.3389/fbioe.2015.00034

**Published:** 2015-03-16

**Authors:** Tabinda Shakeel, Zia Fatma, Tasneem Fatma, Syed Shams Yazdani

**Affiliations:** ^1^Synthetic Biology and Biofuels Group, International Centre for Genetic Engineering and Biotechnology, New Delhi, India; ^2^Department of Biosciences, Jamia Millia Islamia, New Delhi, India; ^3^DBT-ICGEB Centre for Advanced Bioenergy Research, International Centre for Genetic Engineering and Biotechnology, New Delhi, India

**Keywords:** cyanobacteria, marine strain, freshwater strain, hydrocarbon profile, fatty acid synthesis pathway, phylogenetic analysis

## Abstract

The potential utilization of cyanobacteria for the biological production of alkanes represents an exceptional system for the next generation of biofuels. Here, we analyzed a diverse group of freshwater and marine cyanobacterial isolates from Indian culture collections for their ability to produce both alkanes and alkenes. Among the 50 cyanobacterial isolates screened, 32 isolates; 14 freshwater and 18 marine isolates; produced predominantly alkanes. The GC-MS/MS profiles revealed a higher percentage of pentadecane and heptadecane production for marine and freshwater strains, respectively. *Oscillatoria* species were found to be the highest producers of alkanes. Among the freshwater isolates, *Oscillatoria* CCC305 produced the maximum alkane level with 0.43 μg/mg dry cell weight, while *Oscillatoria formosa* BDU30603 was the highest producer among the marine isolates with 0.13 μg/mg dry cell weight. Culturing these strains under different media compositions showed that the alkane chain length was not influenced by the growth medium but was rather an inherent property of the strains. Analysis of the cellular fatty acid content indicated the presence of predominantly C16 chain length fatty acids in marine strains, while the proportion of C18 chain length fatty acids increased in the majority of freshwater strains. These results correlated with alkane chain length specificity of marine and freshwater isolates indicating that alkane chain lengths may be primarily determined by the fatty acid synthesis pathway. Moreover, the phylogenetic analysis showed clustering of pentadecane-producing marine strains that was distinct from heptadecane-producing freshwater strains strongly suggesting a close association between alkane chain length and the cyanobacteria habitat.

## Introduction

The increased carbon footprint and the rapidly depleting petroleum reserves have necessitated the development of responsible, environment-friendly alternatives with immediate impact (Timilsina, 2013). Within this context, the biological production of alkanes, which can serve as drop-in fuels, offers a possible solution. As major constituents of diesel, gasoline, and jet fuels along with their high-energy content and compatibility with the existing fossil fuel infrastructure, alkanes are ideal candidates in this regard (Speight, 1997).

Several prokaryotic and eukaryotic organisms that naturally produce hydrocarbons have been reported in the literature (Coates et al., 2014). The most prominent among these are the cyanobacteria (Winters et al., 1969), which produce both alkanes and alkenes using fatty acid precursors via two different pathways (Schirmer et al., 2010; Mendez-Perez et al., 2011). One pathway involves two terminal enzymes, acyl–acyl carrier protein reductase (AAR) and aldehyde deformylating oxygenase (ADO), which successively convert fatty acyl intermediates to alkanes (Schirmer et al., 2010; Li et al., 2011, 2012; Zhang et al., 2013). Through another pathway, medium chain alkenes (also known as α-olefins) are produced via large multi-domain protein homologous to type I polyketide synthases (PKS). Here, the fatty acyl-ACP is converted to an α-olefin via elongation–decarboxylation mechanism leading to odd-numbered carbon chain with the double bond at the α position (Mendez-Perez et al., 2011). There have been no cyanobacterial species described so far with genomes that harbor both these pathways (Coates et al., 2014). However, it appears from the recent study that the AAR/ADO pathway is prevalent in alkane-producing cyanobacteria (Klähn et al., 2014). In an earlier study, deletion of these two genes rendered cyanobacteria incapable of producing alkanes with no obvious impact on the growth of these cyanobacteria (Schirmer et al., 2010). This suggested that alkane production is not imperative for the survival of these cyanobacteria. However, this observation also raised the question about the relevance of alkane production in cyanobacteria since even after considerable transcriptomic analysis of alkane-producing cyanobacteria under various growth conditions, there was no clear correlation between AAR/ADO expression and the environment (Hernandez-Prieto and Futschik, 2012; Klähn et al., 2014). Moreover, since different chain length alkanes were observed in different cyanobacteria, it is difficult to predict whether the difference in chain length was due to the genetic factors or the diverse climatic condition in which they are naturally found (Schirmer et al., 2010).

Cyanobacteria are found in a variety of habitats (Cameron, 1962; Miller and Castenholz, 2000; Garcia-Pichel et al., 2001), with the most prominent ones being found in marine and freshwater environment (Reed et al., 1984; Nadeau and Castenholz, 2000; Martins et al., 2011). Several useful compounds from marine cyanobacteria and its commercial potential have been well documented (Bloch and Tardieu-Guigues, 2014). However, no systematic study has been reported comparing the alkane and/or alkene production capabilities of marine and freshwater cyanobacterial strains.

In this study, we selected cyanobacterial isolates from Indian marine and freshwater habitats and screened them for the production of alkanes and alkenes. We found that carbon chain lengths correlated well with the natural habitat of that cyanobacterium but not with the cultivation media used in the laboratory. We further compared the intrinsic ability of cyanobacteria to produce different chain length fatty acids with the alkane chain length profile. Finally, we performed a phylogenetic analysis of the 16S rDNA sequences of alkane-producing strains and show a distinct clustering of marine and freshwater cyanobacteria.

## Materials and Methods

### Collection and maintenance of cyanobacterial cultures

#### Source

Indian freshwater cyanobacterial isolates were procured from the National Centre for Culture Collection and Utilization of Blue Green Algae, Indian Agriculture Research Institute (IARI), New Delhi and marine cyanobacterial isolates from National Facility for Marine Cyanobacteria (NFMC), Thiruchirapalli, India. *Synechococcus elongatus* PCC7942 and *S. elongatus* PCC7002 were obtained from Pasteur Culture Collection of Cyanobacteria, France.

#### Culture medium and maintenance

The freshwater strains were cultured in BG-11 medium while the marine isolates were cultured in ASN-III medium (Rippka et al., 1979). Experimental cultures were incubated in identical condition at light intensity 49.5 ± 6.6 μmolm^−2^s^−1^; photoperiod 16:8 h (light: dark); temperature 25–30°C and pH 7.3 ± 0.1 with a salinity preference of 25 ppt in case of marine strains.

### Strain isolation

Strain isolation was achieved via antibiotic treatment and/or dilution plating (Sarchizian and Ardelean, 2010). For antibiotic treatment, the cultures (20 mL each) were incubated for 1 h at 25–30°C in BG-11 or ASN-III medium containing 0.1× LB (tryptone – 1 g/L, yeast extract – 0.5 g/L, NaCl – 1 g/L), followed by the addition of antibiotics – penicillin G (100 μg/ml), streptomycin (50 μg/ml), chloramphenicol (10 μg/ml), and augmentin (100 μg/ml) to each of the culture tubes. The samples were incubated for 24 h in dark, washed with sterile water and then incubated in the respective medium (without antibiotics) in light for 24 h. For isolation via dilution plating, cyanobacterial strains were cultured in their respective medium until the log phase. A small volume of each culture was withdrawn and subjected to washing with sterile water followed by washing with respective sterilized medium. Serial dilutions of the cultures were prepared (dilutions up to 1000 times) and plated on BG-11-agar/ASN-III-agar plates. For both methods, strain purity was checked by growing the strains overnight in LB medium in dark.

### Growth curve of cyanobacteria

The growth characteristics of alkane-producing cyanobacteria were analyzed in liquid culture. Equal amount of samples of freshwater and marine cyanobacterial cultures corresponding to a final concentration of 0.02 mg dry cell weight/mL were inoculated in their respective media in 50 mL volume for each time point and grown for 10 days under optimal growth conditions as mentioned in the previous section. Each 50 mL culture was harvested after an interval of 24 h (up to the tenth day after inoculation) and the samples were centrifuged and dried in the oven at 65°C. Multiple readings were taken during the incubation period until a constant weight was attained by all the samples (Figure S1 in Supplementary Material). The dry weight of each of the samples was measured and normalized per milliliter of the culture. Strain cultivation was performed in triplicate and data plotted as an average. The hydrocarbon estimation was performed for the cultures harvested from day 5 to 9.

### Determination of the alkane and alkene content

For alkane and alkene analysis, the strains were grown in 1 L culture in Erlenmeyer flasks at 25–30°C for 8 days and the hydrocarbons were extracted and analyzed using the protocol modified from Schirmer et al. (2010). Briefly, the sample volume corresponding to 20 mg dry cell weight [cells dried at 65°C until the weight was constant (Figure S1 in Supplementary Material)] was withdrawn from the culture, centrifuged for 1 min at 13,000 rpm, resuspended in methanol and sonicated using a Sonics VibraCell for 10 min at amplitude of 30% and a pulse of 9.9 s on/off. After centrifugation for 3 min at 13,000 rpm, the supernatants were transferred to fresh vials and analyzed on a 7890 A gas chromatography system equipped with a 7000 GC/MS triple quadrapole system (Agilent). The HP-5 capillary column (30 m length, 0.32 mm internal diameter, 0.25 μm film thickness) was used for the separation of metabolites with following method parameters: 1 μL sample was injected (splitless) onto the GC-MS column with inlet temperature at 150°C, the oven was held at 100°C for 3 min. The oven temperature was ramped up to 300°C at a rate of 20°C/min and was then held at 300°C for an additional 5 min. The MS quadrupole scanned from 50 to 550 *m*/*z*. Retention times and fragmentation patterns of product peaks were compared with authentic references to confirm peak identity. The hydrocarbon concentrations were quantified through multiple reaction monitoring (MRM) mode based on commercial standards (Absolute Standards).

### Determination of the fatty acid content

For the analysis of the cellular fatty acid content, cyanobacterial cultures were harvested on day 8. Cell pellets were dried in the oven at 65°C and then crushed using pestle and mortar (Ahmad et al., 2012). The dried sample (100 mg) was used for fatty acids extraction with chloroform and methanol and finally with hexane. Trans-esterification was done using 2 M potassium hydroxide in methanol. The upper hexane layer was collected, filtered, and transferred to fresh vials for fatty acyl methyl ester (FAME) analysis by GC/MS triple quadrapole system. Quantification of FAME was carried out using the 7890 A gas chromatography system equipped with Omega Wax 250 column (30 m length, 0.25 mm internal diameter, 0.25 μm film thickness). The following method parameters were employed: 1 μL sample was injected (split ratio 1:10; inlet temperature held at 150°C) onto the GC column, the oven was held at 140°C for 5 min. The oven temperature was ramped up to 240°C at a rate of 4°C/min and was then held at 240°C for an additional 20 min. Supelco^®^ 37 component FAME mix (Sigma Aldrich) was used as standard to identify the retention times and fragmentation patterns of the product peaks and for quantification of the specific FAME.

### Phylogenetic analysis

Genomic DNA from the cyanobacterial host was isolated by the conventional hexadecyl-trimethyl-ammonium bromide (CTAB) method (Zhang et al., 2014) and was used as template to amplify the 16S rDNA sequence with the help of universal primers 27F (5′-AGAGTTTGATCMTGGCTCAG -3′) and 1492R (5′-TACGGYTACCTTGTTACGACTT-3′). The PCR product was analyzed on a 1% agarose gel in the presence of ethidium bromide, purified using a gel extraction kit (Genetix) and sequenced at Macrogen Inc. The phylogenetic analysis of cyanobacterial strains was conducted in MEGA6 (Tamura et al., 2013) with the help of 16S rDNA sequences using the Neighbor-Joining method (Saitou and Nei, 1987). The bootstrap analysis was performed employing 1000 bootstrap replications (Felsenstein, 1985). The evolutionary distances were computed using the *p*-distance method (Nei and Kumar, 2000).

## Results

### Screening of freshwater and marine cyanobacteria for alkane and alkene production

We obtained 21 freshwater and 29 marine cyanobacterial strains from IARI and NFMC culture collections, respectively (Table 1). Since most of these cultures were contaminated with bacteria, we initially attempted to isolate the cyanobacterial strains via antibiotic treatment. However, many antibiotics interfered with the growth of cyanobacteria and, in a large number of cases, did not reduce the contamination. We therefore resorted to serial dilution to isolate the cyanobacterial strains by transferring four to five times on agar plates. The purity of the cyanobacterial culture was verified by growth on LB liquid medium, LB agar plate, and bright field microscopy.

**Table 1:** **List of cyanobacterial strains analyzed for alkane and alkene production**.

Freshwater strains (IARI culture collection)	Marine strains (NFMC culture collection)
*Anabaena* CCC531	*Aphanocapsa litorallis* BDU130182
*Aulosira* sp. CCC444	*Aphanocapsa* sp. BDHKU35702
*Calothrix* sp. CCC236	*Aphanothece* sp. BDHKU40501
*Chrococcus* CCC429	*Gleocapsa crepidinum* BDHKU10204
*Cylindrospermum* CCC251	*Gleocapsa gigantea* BDU10011
*Hapalosiphon* sp. CCC51	*Lyngbya confervoides* BDU142001
*Lyngbya* sp. CCC473	*Lyngbya* sp. BDU90901
*Nostoc muscorum* CCC92	*Microcoleus acutissimus* BDU140572
*Oscillatoria* sp. CCC305	*Myxosarcina* sp. BDHKU33603
*Oscillatoria* sp. CCC309	*Myxosarcina spectalis* BDU40882
*Phormidium* sp. CCC191	*Oscillatoria boryana* BDU91531
*Phormidium* sp. CCC236	*Oscillatoria formosa* BDU30603
*Phormidium* sp. CCC317	*Oscillatoria jasorensis* BDU51031
*Phormidium* sp. CCC469	*Oscillatoria laetivirens* BDU100891
*Phormidium* sp. CCC470	*Oscillatoria minnesotensis* BDU41121
*Phormidium* sp. CCC495	*Oscillatoria salina* BDU10142
*Plectonema* sp. CCC475	*Oscillatoria willei* BDU141541
*Spirulina platensis* CCC477	*Phormidium angustissimum* BDU100401
*Synechococcus* sp. CCC436	*Phormidium corium* BDU30241
*Tolypothrix* CCC443	*Phormidium fragile* BDU42911
*Westiellopsis* CCC4	*Phormidium* sp. BDUN661
	*Phormidium tenue* BDU40061
	*Phormidium valderianum* BDU40231
	*Plectonema terebrans* BDU141661
	*Pseudanabaena schmidlei* BDU20761
	*Spirulina* sp. BDU51781
	*Spirulina subsalsa* BDU30311
	*Synechococcus elongatus* BDU130192
	*Synechocystis pevaleikii* BDHKU35101

**Figure 1:**
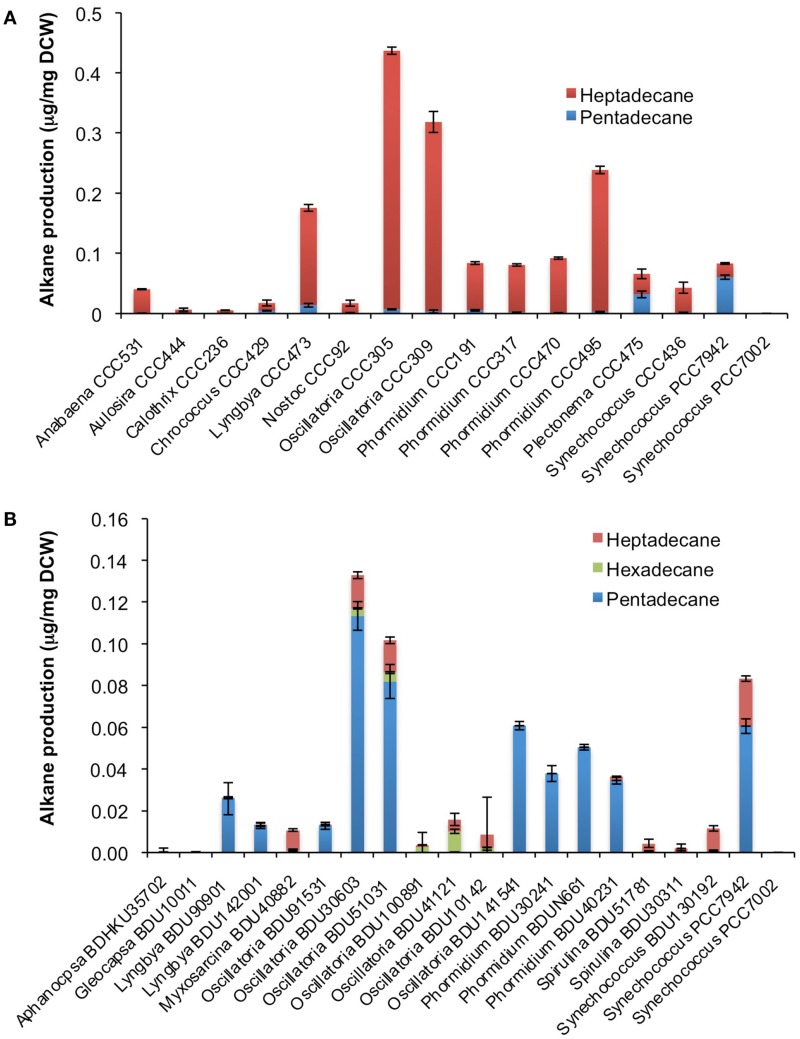
**Alkane profile of freshwater (A) and marine (B) cyanobacterial strains**. Data represent average and SD of three independent experiments.

**Figure 2:**
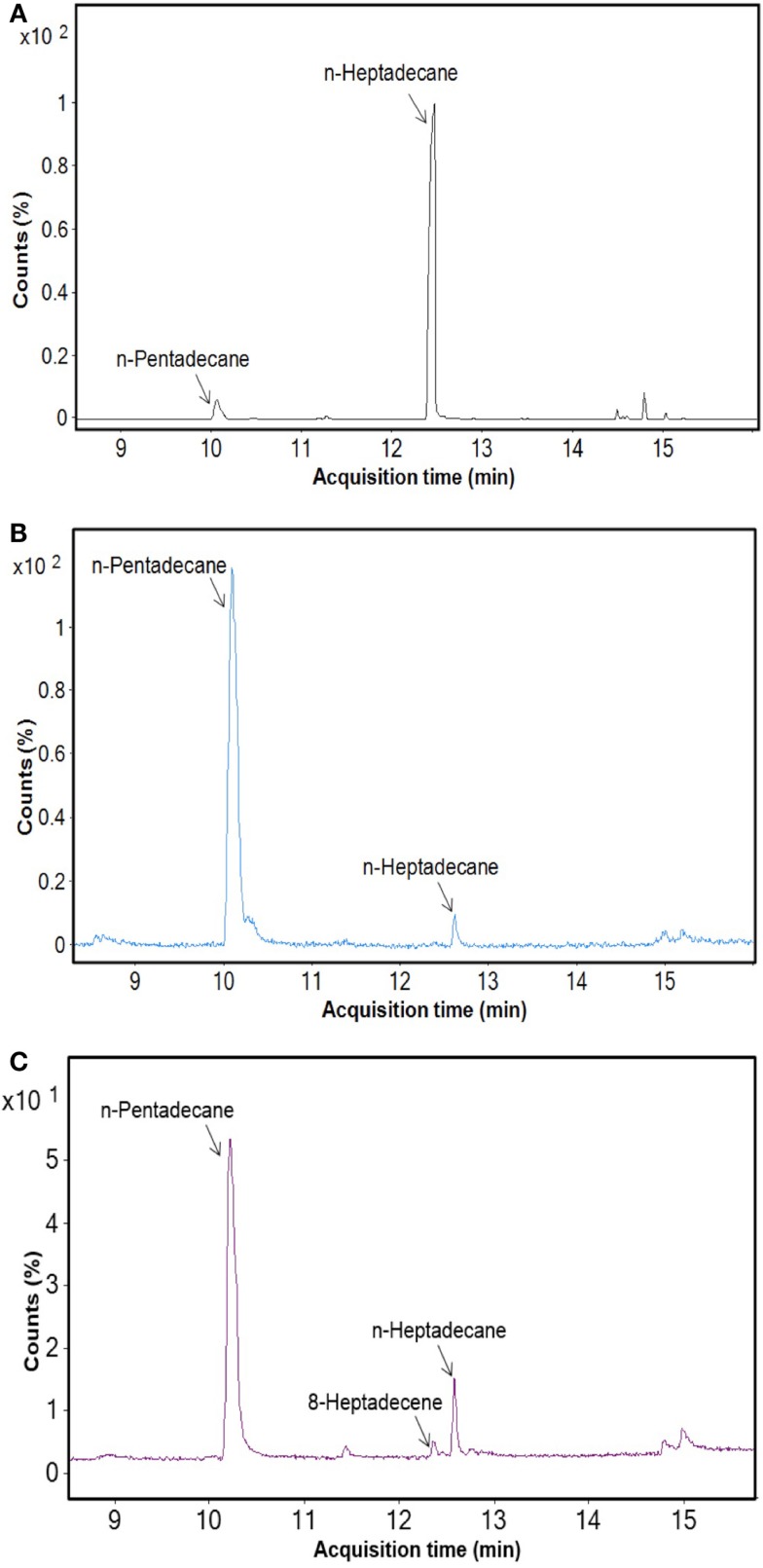
**GC-MS profile of freshwater and marine cyanobacteria**. **(A)** *Oscillatoria* CCC305 strain (freshwater), **(B)** *Oscillatoria formosa* BDU3063 (marine), **(C)** *Synechococcus elongatus* PCC7942 (positive control).

**Figure 3:**
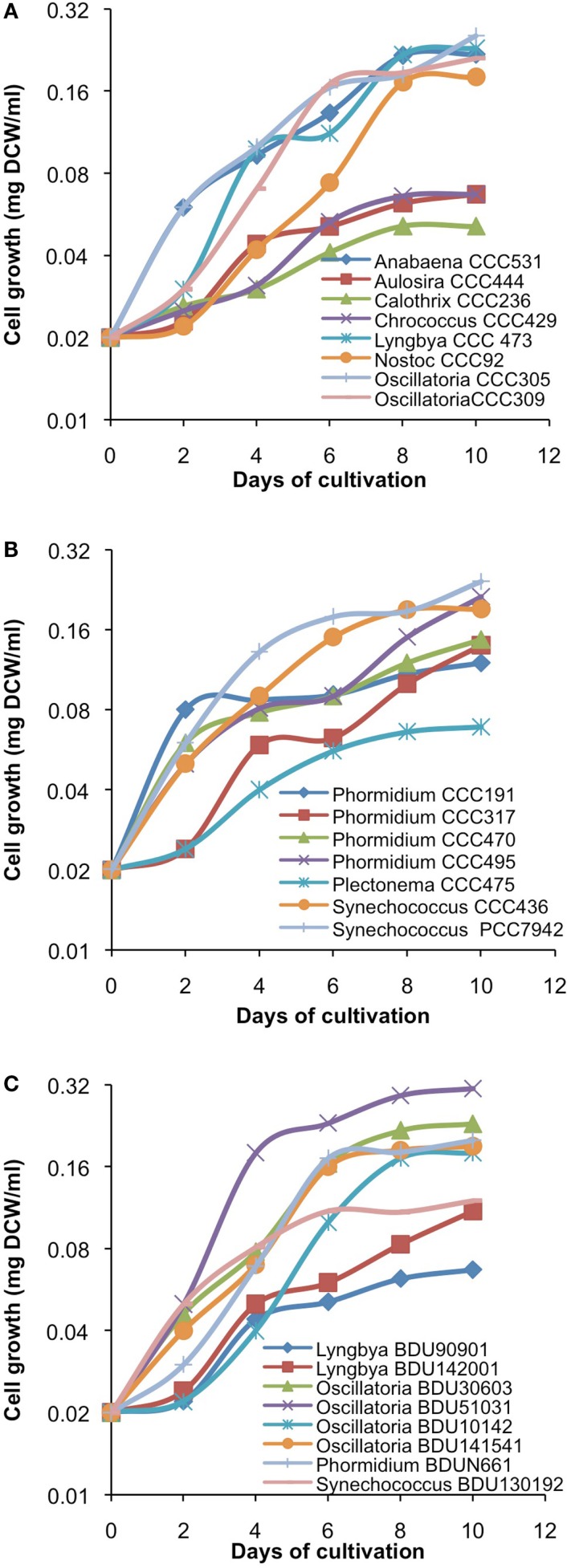
**Growth kinetics of freshwater [(A)-from alphabet A–O] and [(B)-from alphabet P–S] and marine (C) cyanobacterial strains**. Data represent average of three independent experiments.

**Figure 4:**
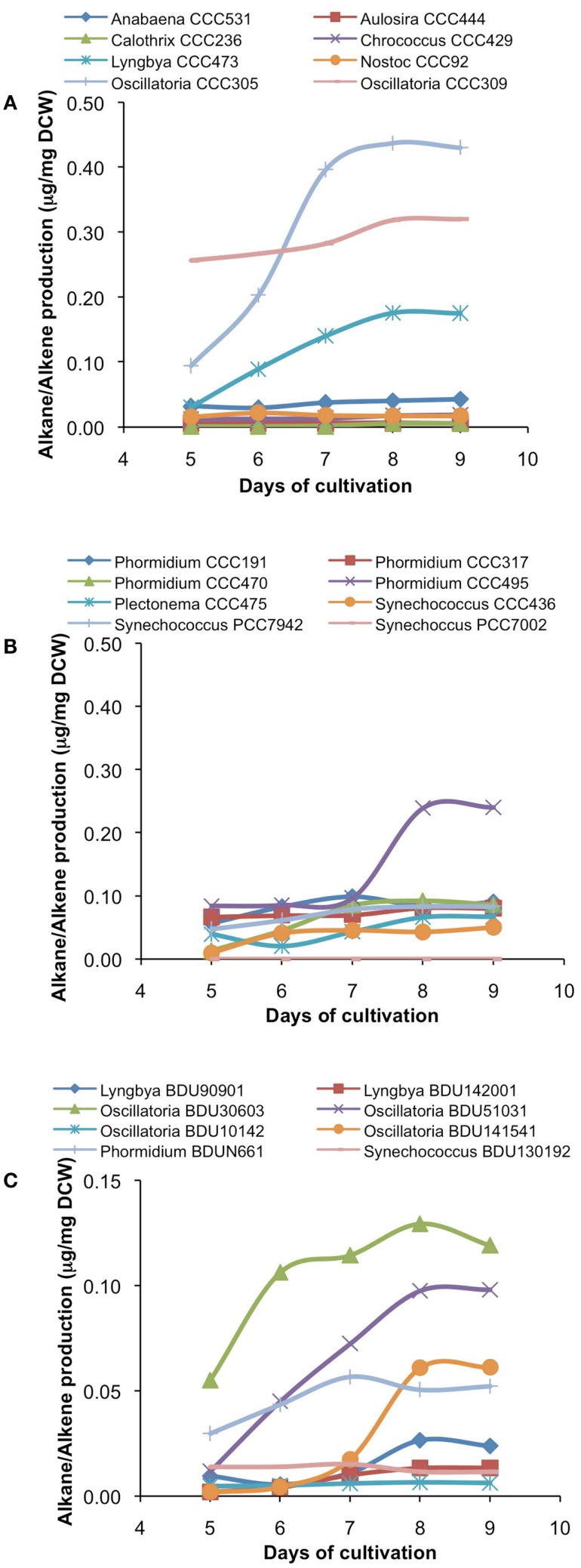
**Alkane/alkene production profile of freshwater [(A)-from alphabet A–O] and [(B)-from alphabet P–S] and marine (C) cyanobacterial strains at different days of growth**. Data represent average of three independent experiments.

After cultivation for 8 days, alkanes and/or alkenes were extracted from both the freshwater and marine strains cultivated in their respective liquid media and analyzed via GC-MS. *S. elongatus* PCC7942 was used as a positive control strain and *S. elongatus* PCC7002 was used as a negative control strain due to the presence and absence of the alkane-producing AAR/ADO pathway, respectively (Schirmer et al., 2010). A total of 32 cyanobacterial strains, which included 14 freshwater and 18 marine strains, were found to produce significant quantities of alkanes though alkenes were also detected (Table S1 in Supplementary Material). We selected these strains and analyzed the chain lengths of the alkanes produced along with their quantification through MRM mode of triple quadrapole mass spectrometer. The majority of these strains produced pentadecane and heptadecane (Figure 1). The freshwater and marine strains predominantly produced heptadecane and pentadecane, respectively, though a few marine stains also produced hexadecane. *Oscillatoria* strains were the highest producers of alkanes for both the freshwater and marine categories. *Oscillatoria* CCC305 produced a total alkane content of 0.43 μg/mg dry cell wt, which was three times higher than the amount produced by the best alkane-producing marine strain *Oscillatoria formosa* BDU30603 (0.13 μg/mg dry cell wt), and more than five times higher than the amount produced by *S. elongatus* PCC7942 (0.083 μg/mg dry cell wt) (Figure 1). The representative plots of the full GC-MS scan and the alkane/alkene quantitation of these strains through MRM are given in Figure 2 and Figure S2 in Supplementary Material, respectively. *Phormidium* sp. were the second highest alkane-producers for both freshwater and marine cyanobacteria. *S. elongatus* PCC7942, a freshwater isolate, produced 0.022 μg/mg dry cell wt pentadecane and 0.0076 μg/mg dry cell wt heptadecane. This was in stark contrast to the other freshwater strains, which predominantly produced heptadecane. As expected, *S. elongatus* PCC7002, which was used as a negative control strain, did not shows any alkane and/or alkene production. Growth curves were obtained for strains, which produced significant quantities of alkane in order to identify optimum levels of alkane production (Figure 3). For most of the strains, the exponential growth phase ranged from 2 to 8 days after which point the growth rate declined. We analyzed alkane production between day 5 and 9 of growth. The trend showed an increase in alkane production until day 8 for most of the strains and then reaching saturation (Figure 4).

### Impact of cultivation media on hydrocarbon production

**Figure 5:**
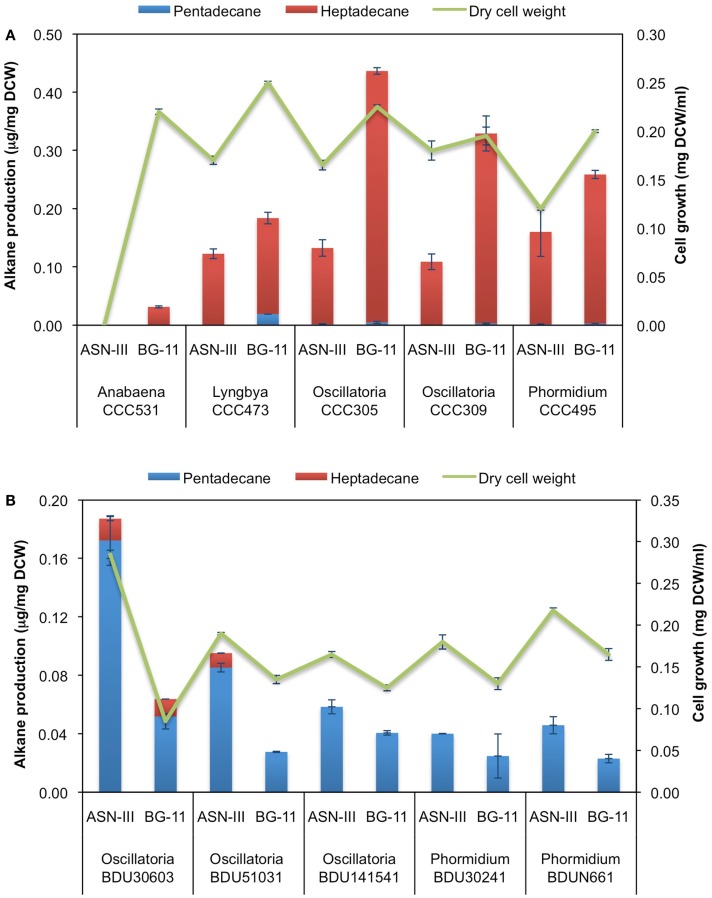
**Impact of growth medium on hydrocarbon profile of (A) freshwater and (B) marine cyanobacterial strains**. Data represent average and SD of three independent experiments.

Based on our GC-MS/MS analyses, we observed that the majority of freshwater cyanobacterial strains produced heptadecane as the predominant alkane while marine strains produced pentadecane. To investigate whether alkane chain length is associated with differences in composition of the cultivation media, we grew five (highest alkane-producers) of the freshwater strains in ASN-III medium, which has high-salt concentration and is generally used to grow marine strains, and five (highest alkane-producers) of the marine strains in BG-11 medium, which has low-salt concentration and is commonly used for growing freshwater strains. *S. elongatus* PCC 7942, classified as a freshwater strain (as indicated on the culture collection website), served as the reference strain and was grown in the ASN-III medium. All strains showed hampered growth due to change in cultivation medium; nevertheless, they grew and attained reasonable biomass at 8 days of cultivation (Figure 5). Upon GC-MS/MS analysis it was found that the freshwater strains predominantly produced heptadecane while the marine strains produced pentadecane, akin to the trend observed with standard cultivation medium (Figure 5). These findings were a clear indication that a change in the media composition from low- to high-salt concentration or vice versa had no major impact on the chain length of the alkanes produced.

### Fatty acid distribution in cyanobacteria

**Figure 6:**
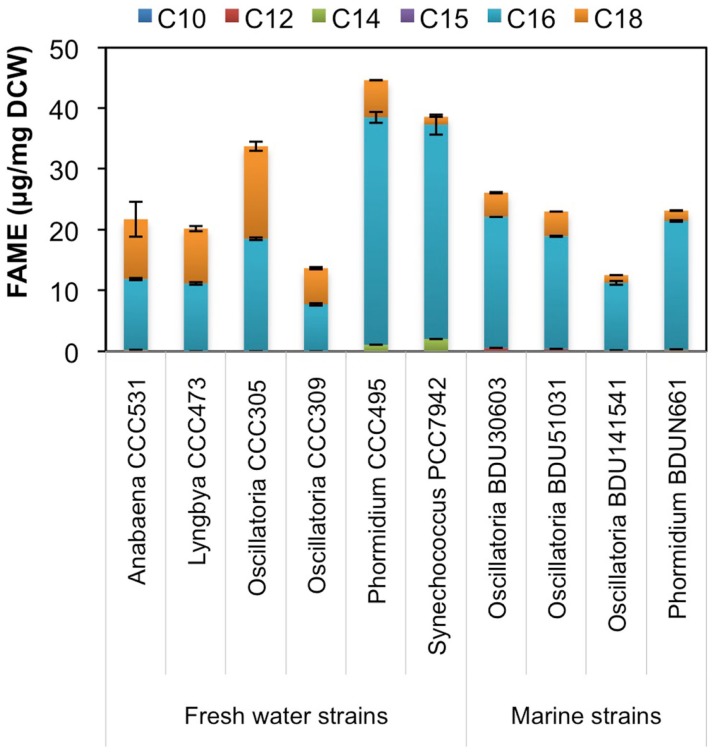
**Fatty acid chain length profile of freshwater and marine cyanobacterial strains**. Cellular fatty acids were extracted from cyanobacteria, trans-esterified to fatty acyl methyl ester (FAME) and analyzed on GC-MS/MS. Data represent average and SD of three independent experiments.

Since environmental factor such as media composition did not contribute to differences in the hydrocarbon chain length in freshwater and marine cyanobacteria, we looked at the possible involvement of fatty acid synthesis pathway in determining the hydrocarbon chain length characteristics. We extracted fatty acids from the major alkane-producing cyanobacterial strains, trans-esterified them and analyzed on GC-MS/MS, as mentioned in Section “Materials and Methods.” We found that the marine strains consistently produced higher amount of C16 chain length fatty acid (Figure 6), which is a precursor for the C15 chain length alkane (pentadecane) (Schirmer et al., 2010), a characteristics for marine strains. On the other hand, the relative proportion of C18 chain length fatty acids increased significantly in freshwater strains as compared to marine, thus explaining the presence of the higher C17 chain length (heptadecane) in these strains. However, *Phormidium* CCC495 produced a much higher quantity of C16 chain length fatty acids though the higher C17 chain length alkane (heptadecane) was still present (Figure 2A). The positive control *S. elongatus* PCC7942 strain continued to show a higher level of C16 in concordance with higher production of pentadecane. An intriguing observation was made with regards to the cellular fatty acid composition of both freshwater and marine cyanobacteria. We found that unsaturated fatty acid contents were significantly higher (Figure S3 in Supplementary Material), which were not reflected in the low abundance of alkenes.

### Phylogenetic analysis of alkane-producing strains

**Figure 7:**
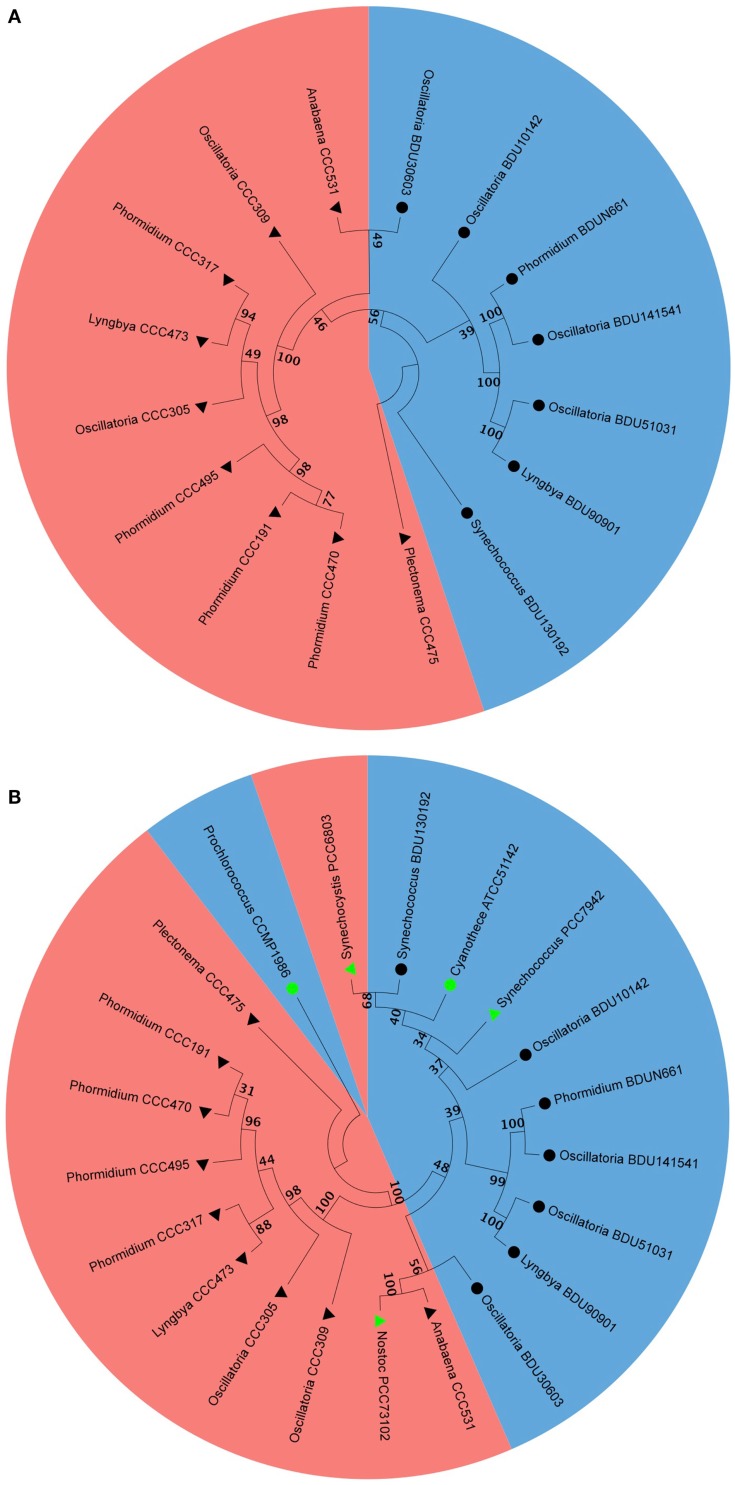
**Phylogenetic analysis of marine and freshwater cyanobacteria from Indian culture collection alone (A) and along with some of the published strains (B)**. Filled triangle represent freshwater, filled circle represent marine, black and green triangles and circles represent cyanobacteria from Indian culture collection and from the published literature, respectively. Blue color and red color shading represent pentadecane and heptadecane producer, respectively.

We selected the alkane-producing cyanobacterial strains and analyzed their phylogenetic properties by aligning the 16S rDNA sequence via Neighbor-Joining method. We found that all marine strains occupied one sub-branch while all freshwater strains occupied another sub-branch (Figure 7A). The branching pattern concurred quite well with alkane chain length, where the pentadecane was associated with the marine sub-branch while heptadecane was associated with the freshwater sub-branch. For this analysis, the 16S rDNA sequences of five cyanobacterial strains reported to be hydrocarbon producers by Schirmer et al. (2010) were included: *Nostoc* PCC73102, *S. elongatus* PCC6803, *S. elongatus* PCC7942, *Prochlorococcus* CCMP1986, and *Cyanothece* ATCC51142. The information available at the culture collection website indicated *Nostoc* PCC73102, *S. elongatus* PCC6803, *S. elongatus* PCC7942 as freshwater strains, and *Prochlorococcus* CCMP1986 and *Cyanothece* ATCC51142 as marine strains. We noted that *S. elongatus* PCC7942 aligned along with pentadecane-producing strains that originated from the marine habitat (Figure 7B). *S. elongatus* PCC6803 also clustered with pentadecane producers, though it was reported to be a heptadecane producer (Schirmer et al., 2010). Others aligned in the clusters as expected from their habitat and carbon chain length characteristics.

## Discussion

Recent characterization of the alkane-producing AAR/ADO pathway in cyanobacteria opens up the possibility of producing alkanes within a commercial context using various synthetic biology tools. Cyanobacteria occupying different niches will quite likely have the ability to produce different kinds of hydrocarbon. Prominent among different niches are freshwater and marine habitats. Cyanobacteria from both these habitats have been reported to produce both alkanes and alkenes. However, a detailed study on the characteristics of the alkanes and alkenes produced by the marine and freshwater cyanobacteria is not available.

Given the richness of microbial diversity in India, we obtained freshwater and marine cyanobacterial strains from two Indian culture collections and studied their alkane-producing capacities (Satyanarayana and Johri, 2005). Among the 50 strains, 32 (14 freshwater and 18 marine) strains produced alkanes and to a lesser extent alkenes. The freshwater strains predominantly produced heptadecane while marine strains predominantly produced pentadecane. This unique observation has never been reported before. Furthermore, the lack of unsaturation at the α carbon position would suggest that the AAR and ADO pathway rather than the PKS-type pathway serves as the main route toward alkane/alkene production, corroborating a previous report that a large number of cyanobacteria possess AAR/ADO pathway (Coates et al., 2014).

It is not really known what triggers alkane production in cyanobacteria. Some studies have indicated that environmental signals might be responsible but no clear conclusion has been drawn (Klähn et al., 2014). It has previously been suggested that media composition, especially salt concentration, may play a role in conferring different alkane chain lengths in freshwater and marine strains. In our case, altering the media composition for freshwater and marine strains had no major impact on the composition of the alkane profile. We considered the metabolic characteristics of the cyanobacteria as another possible determining factor. Alkane production is thought to proceed primarily through the fatty acid synthesis (FAS) pathway followed by reduction and a carbonyl group removal via AAR and ADO enzymes (Schirmer et al., 2010). It was observed previously that although the *S. elongatus* PCC6803 produced only heptadecane, the recombinant *E. coli* carrying AAR and ADO of *S. elongatus* PCC6803 produced a mixture of pentadecane and 8-heptadecene (Schirmer et al., 2010). Similarly, while *S. elongatus* PCC7942 has a preference for the production of pentadecane with some heptadecane in the ratio of ~3:1 (Figure 1), the engineered *E. coli* expressing the AAR and ADO of this strain produced mainly pentadecane and 8-heptadecene (Figure S4 in Supplementary Material) (Schirmer et al., 2010). These results indicated that perhaps AAR and ADO are not responsible for the variation in the chain length of the alkanes observed between freshwater and marine strains. We further analyzed the properties of fatty acid synthesis pathway of freshwater and marine strains by analyzing the fatty acid content of the alkane-producing strains. We found that all the marine strains predominantly produced C16 chain length (Figure 6), which provides the rational for them being the predominant C15 chain alkane producer. The relative proportion of C18 chain fatty acid increased in freshwater strains, again providing rational for predominantly C17 chain alkane production by these strains. Interestingly, both the fatty acid and hydrocarbon chain length of *S. elongatus* PCC7942 exhibit characteristics similar to that of marine species, though it is considered a freshwater species. Overall, these data strongly suggest that alkane chain length is strongly determined by the indigenous fatty acid synthesis pathway of the cyanobacterial strain. Previous study showed clustering of AAR/ADO genes along with a few genes of the fatty acid synthesis pathway, which may indicate a close association of fatty acid and alkane biosynthesis (Klähn et al., 2014). However, we also observed that several fatty acids in these cyanobacteria were unsaturated though this did not correlate with accumulation of the respective alkenes. The unsaturation events were perhaps introduced at a later stage by the known desaturase enzymes, the prominent one being cyanobacterial DesA that introduces double bond at the delta position of fatty acid bound in membrane glycerolipids to provide cold tolerance (Wada et al., 1990). It seems quite possible that alkane synthesis and hydrocarbon chain unsaturation may form diverging pathways from fatty acid synthesis.

Since it was evident from the above observation that genetic factor determines the hydrocarbon chain length in the cyanobacterial strains, we therefore analyzed the phylogenetic properties of alkane-producing strains through alignment of their 16S rDNA sequences. Clustering of pentadecane-producing marine cyanobacteria on one side of the evolutionary branch with heptadecane-producing freshwater strains clustering on another suggests that the alkane chain length perhaps evolved according to the natural habitat in which they lived (Figure 7A). What significance this property holds for the cyanobacteria needs to be analyzed further. We also gathered information from the literature regarding the hydrocarbon chain length, 16S rDNA sequence, and the natural habitat of some of the alkane/alkene-producing cyanobacteria reported by Schirmer et al. (2010). Again, *S. elongatus* PCC7942 clustered along with other pentadecane producers from marine source (Figure 7B), though it is considered a freshwater strain within the literature. *S. elongatus* PCC6803, which has been reported to be a heptadecane producer from freshwater source, surprisingly clustered well with pentadecane-producing marine strains. It is tempting to hypothesize from the phylogenetic analysis that these strains might have evolved from the marine environment. While PCC7942 still retains its pentadecane-producing ability, PCC6803 may possibly have switched to heptadecane production. This hypothesis would require further investigation. With a few exceptions, our analysis does suggest that the majority of alkane-producing cyanobacterial strains cluster according to their niche area of habitation and alkane chain length.

In summary, we screened and identified cyanobacterial strains that produced more than fivefold higher alkane than the amount produced by previously reported *S. elongatus* PCC7942. We found that the freshwater strains and marine strains predominantly produced heptadecane and pentadecane, respectively, which correlated well with their phylogenetic clustering. Most importantly, the distinct alkane chain length between freshwater and marine strains is most probably attributed to the subtle differences in the metabolic characteristics of the fatty acid synthesis pathway.

## Author Contributions

SY designed the study. SY and TF supervised the research. TS performed all the experiments related to cyanobacterial hydrocarbon, fatty acid, and phylogenetic analysis. ZF cloned and expressed hydrocarbon pathway enzymes in *E. coli*. SY and TS wrote the manuscript.

## Conflict of Interest Statement

The authors declare that the research was conducted in the absence of any commercial or financial relationships that could be construed as a potential conflict of interest.

## Supplementary Material

The Supplementary Material for this article can be found online at http://journal.frontiersin.org/article/10.3389/fbioe.2015.00034
