# Supplementary material for: Heterogeneity of Alkane Chain Length in Freshwater and Marine Cyanobacteria
Source: Front Bioeng Biotechnol. 2015 Mar 16;3:34. doi: 10.3389/fbioe.2015.00034 (PMC4360714; doi:10.3389/fbioe.2015.00034)
Supplement: Supplementary file 1 [file data_sheet_1.zip › Figure S1.pdf]

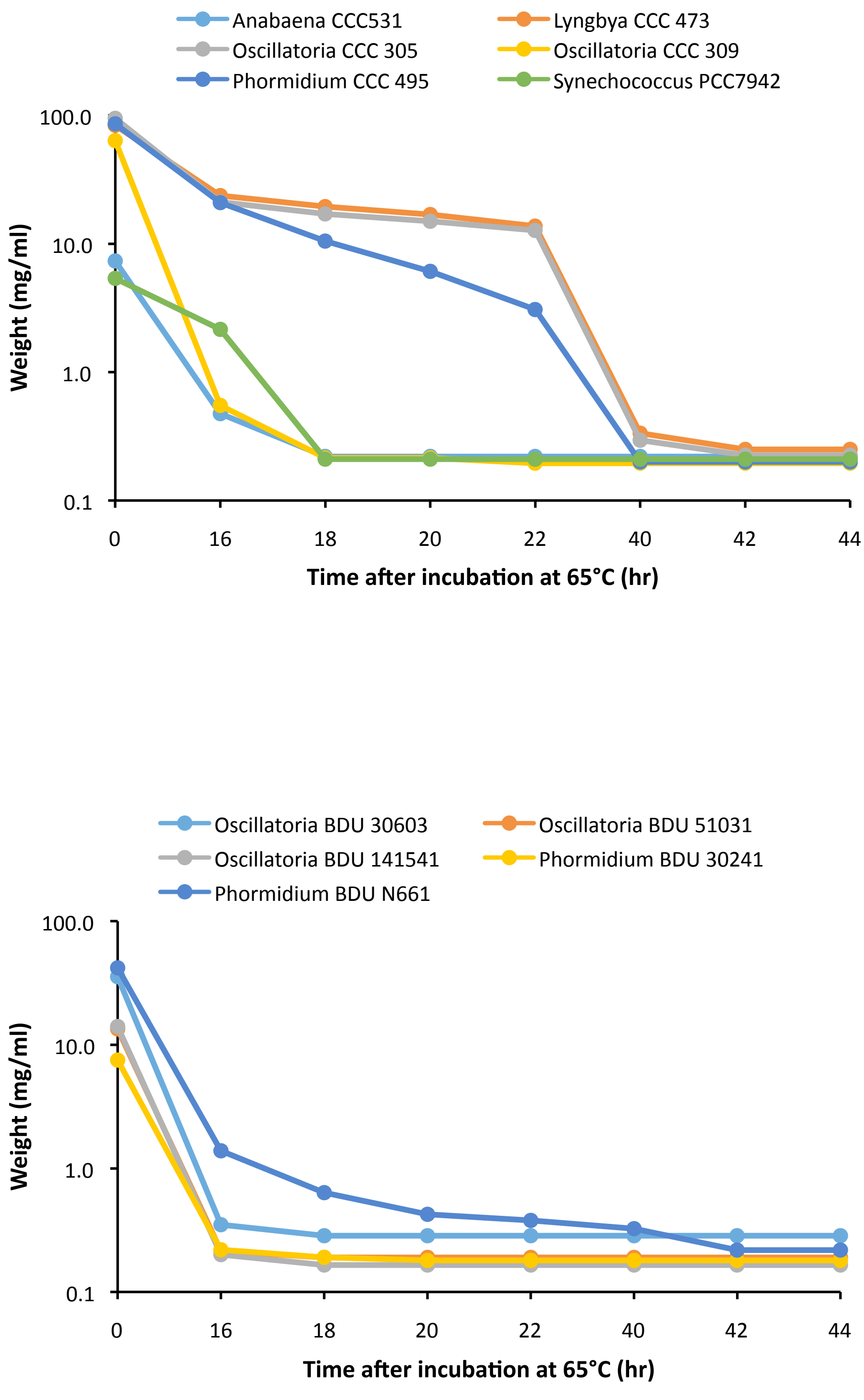

Supplementary Figure 1. Weight change analysis of (A) freshwater and (B) marine strains upon drying at 65 °C.
