# Supplementary material for: Heterogeneity of Alkane Chain Length in Freshwater and Marine Cyanobacteria
Source: Front Bioeng Biotechnol. 2015 Mar 16;3:34. doi: 10.3389/fbioe.2015.00034 (PMC4360714; doi:10.3389/fbioe.2015.00034)
Supplement: Supplementary file 1 [file data_sheet_1.zip › Figure S2.pdf]

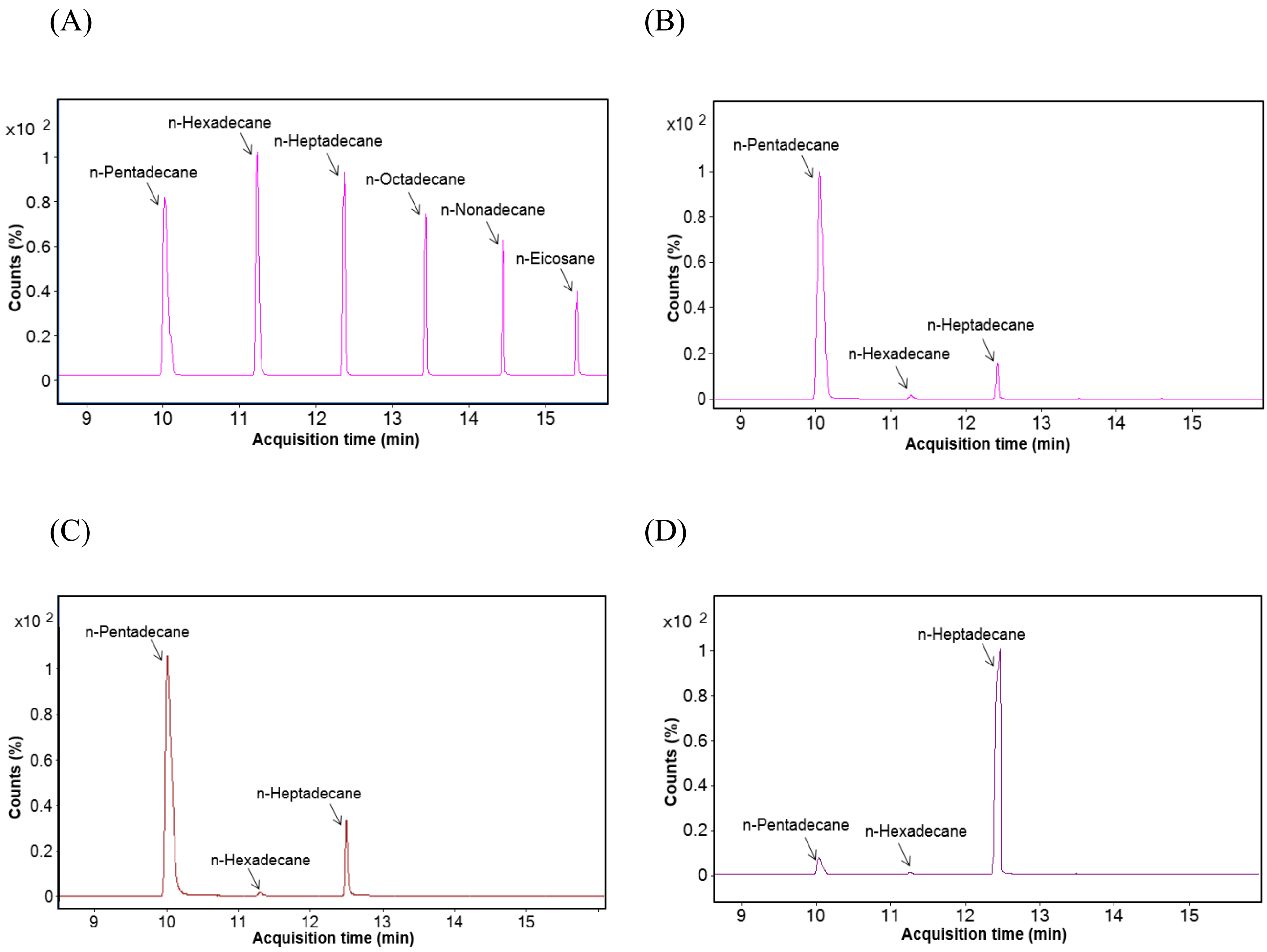

Supplementary Figure 2. GC-MS/MS profiles of (A) Alkanes standard, (B) *Synechococcus elongatus* PCC 7942, (C) *Oscillatoria formosa* BDU3063, and (D) *Oscillatoria* CCC 305
