# Supplementary material for: Heterogeneity of Alkane Chain Length in Freshwater and Marine Cyanobacteria
Source: Front Bioeng Biotechnol. 2015 Mar 16;3:34. doi: 10.3389/fbioe.2015.00034 (PMC4360714; doi:10.3389/fbioe.2015.00034)
Supplement: Supplementary file 1 [file data_sheet_1.zip › Figure S3.pdf]

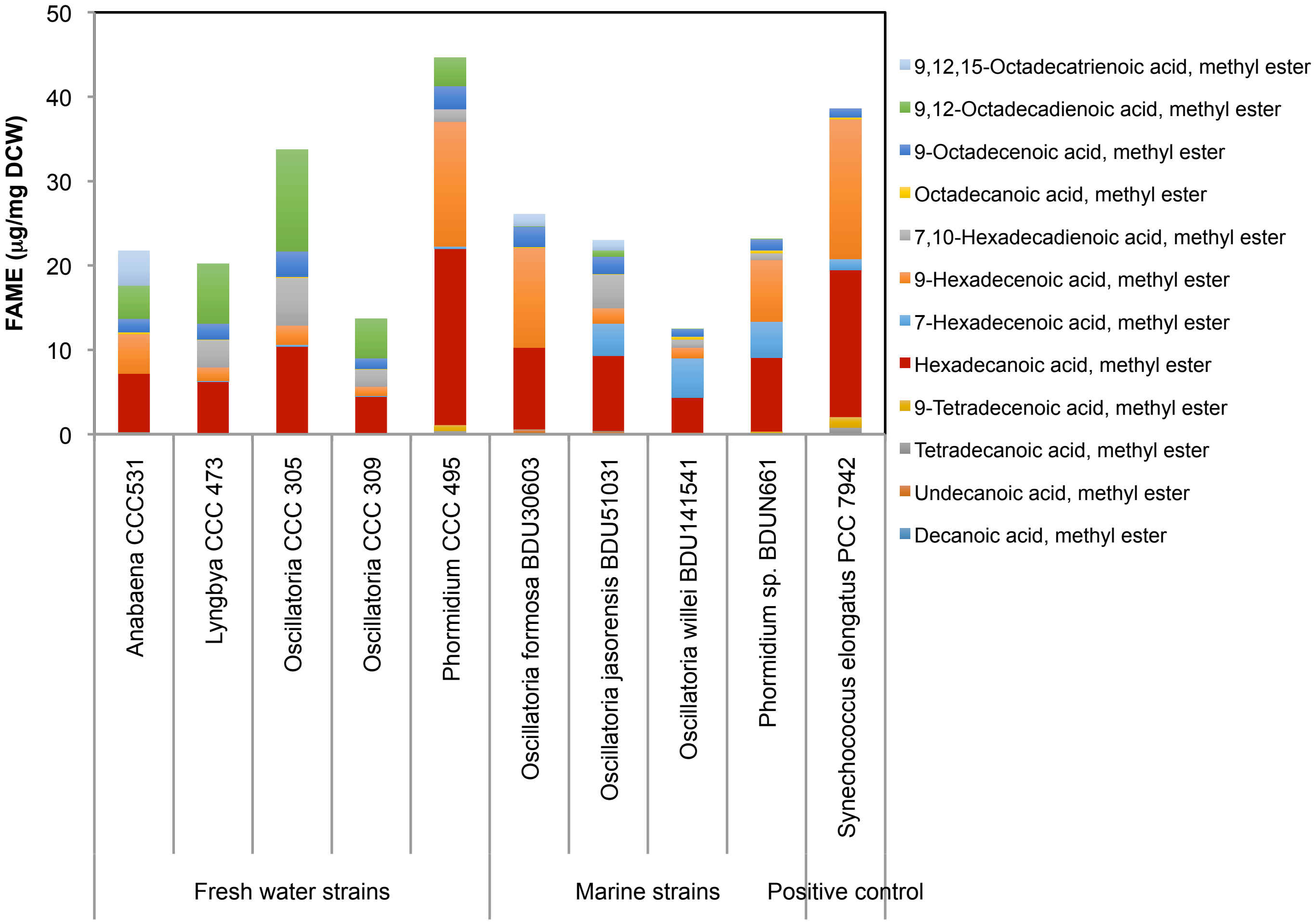

Supplementary Figure 3. Cellular fatty acids chain length profile of hydrocarbon producing marine and fresh water cyanobacteria along with their saturation/unsaturation. Cellular fatty acids were extracted from cyanobacteria, transesterified to fatty acyl methyl ester (FAME) and analyzed on GC-MS/MS.
