# Supplementary material for: Heterogeneity of Alkane Chain Length in Freshwater and Marine Cyanobacteria
Source: Front Bioeng Biotechnol. 2015 Mar 16;3:34. doi: 10.3389/fbioe.2015.00034 (PMC4360714; doi:10.3389/fbioe.2015.00034)
Supplement: Supplementary file 1 [file data_sheet_1.zip › Figure S4.pdf]

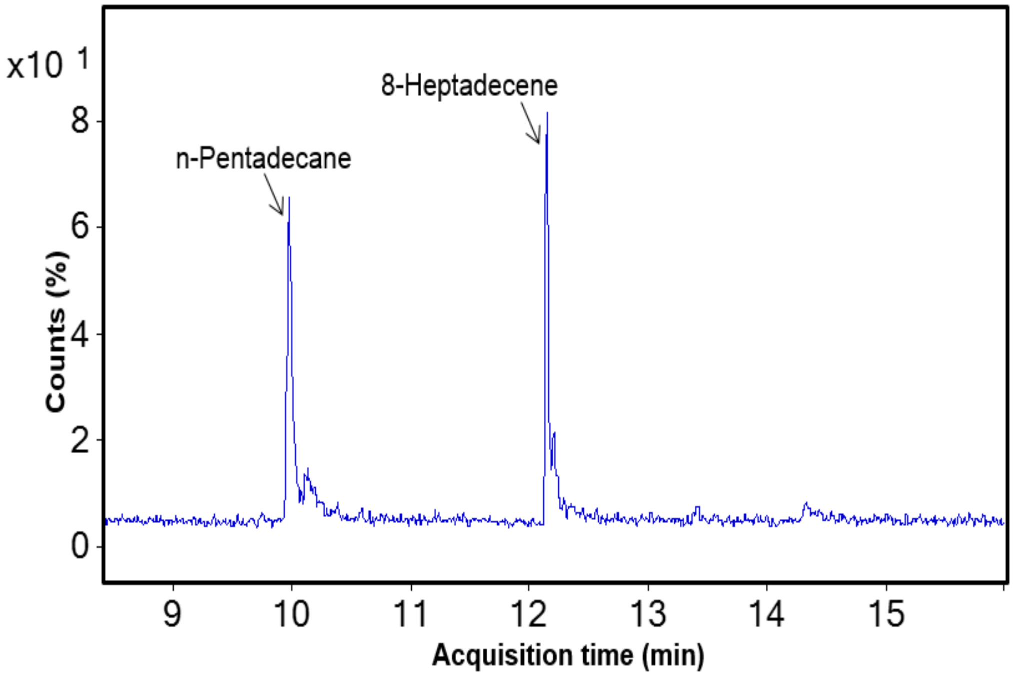

Supplementary Figure 4. GC-MS profile of hydrocarbon produced by recombinant *E. coli* carrying genes for acyl ACP reductase (AAR) and aldehyde deformylating oxygenase (ADO). Genes for AAR and ADO were codon optimized, cloned in pQE30 plasmid and expressed in *E. coli* DH5a. The hydrocarbon of the grown and induced cells were extracted and analyzed on GC-MS/MS.
