# Supplementary material for: Heterogeneity of Alkane Chain Length in Freshwater and Marine Cyanobacteria
Source: Front Bioeng Biotechnol. 2015 Mar 16;3:34. doi: 10.3389/fbioe.2015.00034 (PMC4360714; doi:10.3389/fbioe.2015.00034)
Supplement: Supplementary file 1 [file data_sheet_1.zip › Table S1.docx]

**Supplementary Table 1.** Detection of alka(e)nes in the fresh water and marine cyanobacterial isolates

| **S. No.** | **Cyanobacterial strain** | **Alka(e)ne chain lenght^a^** | | |
| --- | --- | --- | --- | --- |
| **Fresh water isolates** | | C15 | C16 | C17 |
| 1 | *Anabaena* CCC531 | + | +/- | + |
| 2 | *Aulosira sp.* CCC444 | + | - | + |
| 3 | *Calothrix sp.* CCC236 | + | - | + |
| 4 | *Chrococcus* CCC429 | + | +/- | + |
| 5 | *Cylindrospermum* CCC251 | +/- | - | +/- |
| 6 | *Hapalosiphon sp.* CCC51 | - | - | +/- |
| 7 | *Lyngbya sp.* CCC473 | + | +/- | + |
| 8 | *Nostoc muscorum CCC92* | + | +/- | + |
| 9 | *Oscillatoria sp.* CCC305 | + | +/- | + |
| 10 | *Oscillatoria sp.* CCC309 | + | +/- | + |
| 11 | *Phormidium sp.* CCC191 | + | - | + |
| 12 | *Phormidium sp.* CCC236 | - | - | +/- |
| 13 | *Phormidium sp.* CCC317 | + | - | + |
| 14 | *Phormidium sp.* CCC469 | - | - | +/- |
| 15 | *Phormidium sp.* CCC470 | + | - | + |
| 16 | *Phormidium sp.* CCC495 | + | - | + |
| 17 | *Plectonema sp.* CCC475 | + | - | + |
| 18 | *Spirulina platensis* CCC477 | +/- | +/- | +/- |
| 19 | *Synechococcus sp.* CCC436 | + | - | + |
| 20 | *Tolypothrix* CCC443 | - | - | +/- |
| 21 | *Westiellopsis* CCC4 | - | - | +/- |
| 22 | *Synechococcus elongatus* PCC7942 ^b^ | + | - | + |
| 23 | *Synechococcus elongatus* PCC7002 ^c^ | - | - | - |
| **Marine isolates** | |  |  |  |
| 24 | *Aphanocapsa litorallis* BDU130182 | - | - | - |
| 25 | *Aphanocapsa sp.* BDHKU35702 | + | + | - |
| 26 | *Aphanothece sp.* BDHKU40501 | +/- | - | +/- |
| 27 | *Gleocapsa crepidinum* BDHKU10204 | + | - | + |
| 28 | *Gleocapsa gigantea* BDU10011 | +/- | - | +/- |
| 29 | *Lyngbya confervoides* BDU142001 | + | - | + |
| 30 | *Lyngbya sp.* BDU90901 | + | - | + |
| 31 | *Microcoleus acutissimus* BDU140572 | - | - | +/- |
| 32 | *Myxosarcina sp.* BDHKU33603 | - | - | - |
| 33 | *Myxosarcina spectalis* BDU40882 | + | + | + |
| 34 | *Oscillatoria boryana* BDU91531 | + | - | + |
| 35 | *Oscillatoria formosa* BDU30603 | + | + | + |
| 36 | *Oscillatoria jasorensis* BDU51031 | + | + | + |
| 37 | *Oscillatoria laetivirens* BDU100891 | - | + | + |
| 38 | *Oscillatoria minnesotensis* BDU41121 | + | + | + |
| 39 | *Oscillatoria salina* BDU10142 | + | + | + |
| 40 | *Oscillatoria willei* BDU141541 | + | - | - |
| 41 | *Phormidium angustissimum* BDU100401 | +/- | - | - |
| 42 | *Phormidium corium* BDU30241 | + | - | - |
| 43 | *Phormidium fragile* BDU42911 | +/- | - | - |
| 44 | *Phormidium sp.* BDUN661 | + | - | - |
| 45 | *Phormidium tenue* BDU40061 | +/- | +/- | +/- |
| 46 | *Phormidium valderianum* BDU40231 | + | - | + |
| 47 | *Plectonema terebrans* BDU141661 | +/ | +/- | +/- |
| 48 | *Pseudanabaena schmidlei* BDU20761 | - | - | +/- |
| 49 | *Spirulina sp.* BDU51781 | + | + | + |
| 50 | *Spirulina subsalsa* BDU30311 | + | + | + |
| 51 | *Synechococcus elongatus* BDU130192 | + | - | + |
| 52 | *Synechocystis pevaleikii* BDHKU35101 | +/- | - | +/- |

^a^ (+) : hydrocarbon present at a concentration ≥ 0.0001μg/mg dry cell weight; (+/-) : hydrocarbon present at <0.0001μg/mg dry cell weight; (-) : hydrocarbon absent

^b^ Used as positive control for hydrocarbon production

^c^ Used as negative control for hydrocarbon production
